# A novel model for studying ileitis-induced visceral hypersensitivity in goats

**DOI:** 10.1186/s13028-016-0253-0

**Published:** 2016-10-06

**Authors:** Adnan Hassan Tahir, Juan Wan, Manoj Kumar Shah, Habibullah Janyaro, Xiao-Jing Li, Ming-Xing Ding

**Affiliations:** College of Veterinary Medicine, Huazhong Agricultural University, No. 1, Shizishan Street, Hongshan District, Wuhan, Hubei 430070 People’s Republic of China

**Keywords:** Inflammatory bowel disease, Johne’s disease, Ileitis, TNBS, Visceral hypersensitivity, Visceromotor response

## Abstract

**Background:**

Visceral hypersensitivity (VH) is a common condition in many gastrointestinal disorders such as inflammatory bowel diseases (IBDs) in human and animals. Most studies often induce Crohn’s disease/colitis to investigate VH in small experimental animals. Although farm animals commonly suffer from IBDs, their VH has not been investigated so far. Because goats can suffer from Johne’s disease, a naturally occurring Crohn’s-like disease, they may be suitable to be used for studying the mechanism underlying VH in common intestinal disorders of large animals. In the present study, 60 healthy goats of either sex were equally divided into a 2, 4, 6-trinitrobenzenesulfonic acid (TNBS) group and saline group. A volume of 1.2 ml of TNBS-ethanol solution (30 mg TNBS in 40 % ethanol) or an equal volume of isotonic saline was injected into the wall of the terminal ileum through laparotomy. The severity of the developing ileitis was determined according to macro- and microscopic pathologic scores and the levels of myeloperoxidase, interleukin-1β, interleukin-6 and tumor necrosis factor-α, and VH was evaluated with visceromotor responses (VMR) to colorectal distension on days 3, 7, 14, 21 and 28. VMRs were assessed with a continuous ramp distention mode with 6 s for each pressure (20, 40, 60, 80 and 100 mmHg).

**Results:**

Compared to the saline group, the TNBS-treated goats showed apparent transmural pathological changes and a significant increase (*P* < 0.05) in macroscopic and microscopic change scores, and levels of myeloperoxidase, interleukin-1β, interleukin-6 and tumor necrosis factor-α in the ileum, and VMR to colorectal distension. The goats exhibited apparent ileitis at days 3 to 21, and VH at days 7 to 28 following TNBS treatment.

**Conclusion:**

This experiment successfully established a reproducible ileitis and VH with administration of TNBS-ethanol solution in the ileal wall of goats. This model is useful for studying the pathogenesis of the IBD and the mechanism underlying VH, and for evaluating the efficacy of new therapeutic regimens.

## Background

Inflammatory bowel diseases (IBDs) are a collection of chronic inflammatory disorders of the gastrointestinal tract in human and animals. The major symptoms are visceral hypersensitivity (VH) (visceral pain, altered bowel movement, increased mucosal secretion) with weight loss, malnutrition, fever and lack of appetite [1, 2]. VH is one of the important symptoms in about 50–70 % of patients experiencing the initial onset of IBD.

Johne’s disease (JD), a kind of IBDs, is seen in cattle, sheep and goats and is caused by *Mycobacterium avium* subsp. *paratubersulosis*. Gross lesions of JD include intense reddening, mucosal granulomatous inflammation and wall thickening, especially in ileum [3]. This chronic intestinal disease occurs worldwide and causes mortality and productivity loss of ruminants [4, 5]. JD shares many similarities with human Crohn’s disease (CD) in terms of symptoms, location and pathological changes. The small intestine, especially the terminal ileum, is the primary location of both diseases [6, 7]. VH is an important characteristic of IBD in humans, but VH in ruminants is rarely reported. Studies have indicated that VH may result from a dysregulated mucosal immunologic response to one or more antigens present in the enteric flora as well as a genetic predisposition to the development of this response [8–11]. However, its underlying mechanisms are still unknown. Most studies have investigated VH utilizing colitis models; however, such models are not appropriate for studying the exact mechanism by which ileitis induces VH because its location, duration, microbiota and central regulating mechanism are different.

Different chemicals such as acetic acid, formalin, indomethacin, 2, 4, 6-trinitrobenzenesulfonic acid (TNBS), carrageenan and dextran sodium sulfate have been used to study IBD. Among these chemicals, TNBS is the most commonly used. Nowadays, TNBS in combination with ethanol has been used to induce ileitis or colitis in mice [12], rats [13], guinea pigs [14, 15] and pigs [16]. Several studies reported that intracolonic administration of TNBS-ethanol solution can provoke VH in rats [17–19]. So far no VH model of farm animals has been reported.

In the present study, a TNBS-ethanol solution was injected into goat’s terminal ileum wall to induce ileitis and VH. The macroscopic and microscopic damage scores, and levels of myeloperoxidase (MPO) and cytokines were detected for evaluating the severity of the induced inflammation. Visceromotor response (VMR) to colorectal distension (CRD) was used to assess VH.

## Methods

### Experimental animals

Goats were thoroughly examined to ascertain their health status. The animals were kept under the same nutritional and managemental conditions. They were dewormed and acclimatized to the environment for 1 week before the initiation of the study. The experiment was approved by the Institutional Animal Care and Use Committee of Huazhong Agricultural University, Wuhan, China (HZAUSH-2015-007), and performed with the guidelines of the Committee for Research and Ethical Issues of the International Association for the Study of Pain.

### Determination of TNBS dosage for model experiment

Eighteen male goats (about 1-year-old) were randomly divided into 6 groups i.e. saline, 40 % ethanol, 30 mg TNBS (Sigma Aldrich company, USA) and TNBS-ethanol solutions (20, 30 or 40 mg in 40 % ethanol), with 3 goats each group. The goats were kept off feed for 12 h prior to the experiment to avoid anaesthesia-induced regurgitation and other respiratory complications. Baseline cardinal parameters like respiratory rate, pulse rate and body temperature were recorded. The animals were premedicated with atropine (0.03 mg/kg, IM), etamsylate (0.02 g/kg, IM) and xylazole (0.10 mg/kg, IM, North China Pharmaceutical Co., Ltd, Hubei, China). Goats were placed on the left lateral recumbency. After the trachea was intubated to avoid aspiration of ruminal contents, anaesthesia was induced via intravenous administration of ketamine HCl (0.5 mg/kg/min, Yao Pharma Co., Ltd, China). The anaesthetic depth was judged according to eyelid and corneal reflexes and painful response to the prickle of the abdominal skin and coronary hooves. A 6-cm incision was performed on the right flank abdomen following aseptic surgical principle, and the distal part of the ileum was identified and exteriorized. Total 1.2 ml of saline, 40 % ethanol, 30 mg TNBS or TNBS-ethanol solutions (20, 30 and 40 mg in 40 % ethanol) was injected into the ileal wall through five points, approximately 15 cm proximal to the ileocecal junction, with a 30-gauge needle. The intestine was then returned to the abdominal cavity. The abdominal wall and peritoneum were closed using standard procedure. The goats were monitored till recovered from anaesthesia. Tramadol hydrochloride (5 mg/kg, IM) was injected to relieve postoperative pain for 2 days. The wound was treated with 1 % povidone iodine solution daily until completely healed. The animals were resubjected to a similar laparotomy on day 7. The ileal mesentery arteries and veins supplying the injected region were ligated with 3-0 chromic catgut. Four intestinal clamps were applied on both sides of the injected part to prevent content leakage. The 6 cm terminal ileum of each goat was excised 15 cm proximal to the ileocecal junction. The ileum segment was flushed with phosphate buffered saline, cut longitudinally and placed flatly on a clean drape sheet. After detection of the macroscopic changes, a 2 × 2 cm tissue block was taken from the approximate part of the ileum segment opposite to the mesentery, and stored in 10 % formalin for histopathological examination. An expert surgical team performed enteric end-to-end anastomosis immediately after the removal of the partial ileal segment and closed the abdomen using standard methods. The goats received tramadol hydrochloride (5 mg/kg, IM) for 3 days and ampicillin (20 mg/kg, IM) for 5 days. After 2 week recovery, the animals were sent to goat farms. The ileitis severity was determined according to gross and microscopical lesions, and the dose of TNBS inducing moderate ileitis was chosen for the model experiment.

### Experimental setup

**Fig. 1:**
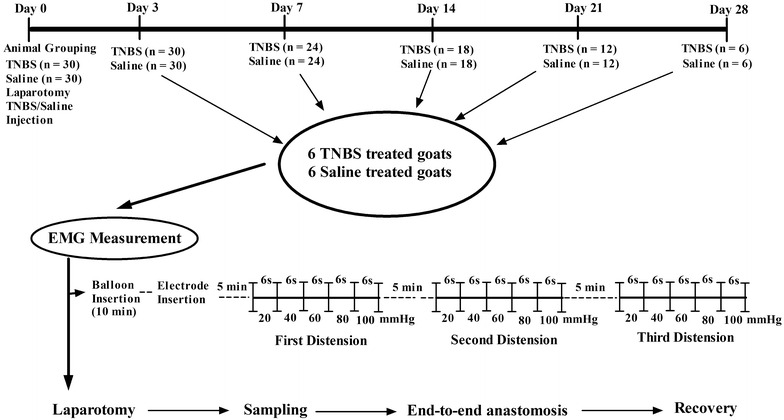
Illustration of the experimental design and electromyography protocol

The experimental setup is illustrated in Fig. 1. Sixty goats (30 males and 30 females, about 1-year-old) were included. The animals were randomly divided into two equal groups receiving TNBS and isotonic saline, respectively. The goats were anesthetized and laporatomized with the methods described above. The distal part of the ileum was exteriorized. For the TNBS group, 1.2 ml of TNBS-ethanol solution (30 mg TNBS in 40 % ethanol) was injected into the ileal wall. In the saline group, the same volume of isotonic saline was injected in the same manner. Animals were weighed at days 0, 3, 7, 14, 21 and 28 following surgery. Six animals were selected from each group at days 3, 7, 14, 21 and 28, respectively, for electromyography (EMG). After EMG, the goats immediately underwent the same laparotomy. The ileum segment was handled as described above. After macroscopic changes were detected, a 2 × 2 cm tissue block was taken from the approximate part of the segment opposite to the mesentery, and preserved in 10 % formalin for histopathological examination. Another 2 × 2 cm tissue block was taken from the distal part opposite to the mesentery, weighed, frozen in liquid nitrogen and finally transferred to store in −80 °C for the measurements of tumor necrosis factor-alpha (TNF-α), interleukin-1 beta (IL-1β), interleukin-6 (IL-6) and MPO. Enteric end-to-end anastomosis and postoperative care were conducted as before.

### Colorectal distension testing

VH was reflected by EMG. EMG was used to record VMR to CRD of goats at days 3, 7, 14, 21 and 28 after surgery as described previously [20]. Briefly, the goats were restrained to keep standing. A manual distension device was made using a T-connector connecting a balloon, a vacuum pump and a sphygmomanometer. The polyethylene balloon (12 cm) was lubricated with paraffin and inserted into the distal colon 10 cm from the anus of goats 10 min before each VMR measurement. Two nickel-steel needles (0.45 mm in diameter and 5 cm in length) were inserted 2 cm apart directly into the external oblique muscle at the center of the left abdomen as electrodes 5 min before each VMR. The electrodes were clamped with adjacent skin, respectively, and connected to an EMG recorder (Nanjing Medease Science and Technology Co., Ltd, China). EMG was amplified and filtered with a processing system MedLab-U/4C501H (Nanjing Medease Science and Technology Co., Ltd, China). The balloon was inflated with the pump. The pressure in the balloon was measured with the sphygmomanometer. In our pretest, goats showed VMR changes when distention pressure increased from 20 to 100 mmHg, and exhibited stronger painful response when pressure reached to 120 mmHg. Therefore, graded pressures (20, 40, 60, 80 and 100 mmHg) were chosen for the model experiment. The balloon was inflated in a continuous ramp distension mode (20, 40, 60, 80 and 100 mmHg). EMG at each stage was recorded for 6 s. The procedure of balloon distension and EMG recordings was repeated every 5 min for 3 times (Fig. 1). Average EMG values for 3 times were analyzed with MedLabV6.3 software (Nanjing Medease Science and Technology Co., Ltd, China) and expressed as millivolt per second (mV/s).

### Macroscopic and microscopic observations

The macroscopic lesions were scored by two independent observers who were unaware of the treatments according to a scale of 0–10 (Table 1). The macroscopic scores were assessed according to adhesion of ileal serosum to the intestinal loops, and mucosal pseudomembrane, hyperemia, ulceration, and wall thickening with a modification of the criteria described by Vadilla et al. [21]. Samples were processed for the histological studies by routine techniques. Six series of 5 µm tissue sections were obtained. Three sections were stained with hematoxylin-eosin (HE). Another 3 were stained with 0.5 % toluidine blue working solution for 3–5 min. Two pathologists were blindly assigned to examine and scored all sections according to crypt depth, inflammatory cells infiltration, submucosal thickness and blood vessel congestion with a modification of the method described previously [21].

**Table 1:** Morphological criteria for the assessment of ileal damage

Macroscopic changes		Scores	Microscopic changes		Scores
Adhesions	None	0	Crypt depth	Normal	0
	Minimum	1		<50 % reduction	1
	Involving several bowel loops	2		>50 % reduction	2
Mucosal hyperemia	Normal	0	Inflammatory cells	No infiltration	0
	Mild	1		Few scattered cells	1
	Moderate	2		Distributed but not dense	2
	Severe	3		Dense	3
Ulcers	None	0	Blood vessel congestion	Normal	0
	Ulceration <2 cm length	1		Mild	1
	Two ulcers <2 cm	2		Moderate	2
	More sites of ulceration or one >2 cm	3		Severe	3
Wall thickness	Normal	0	Ulceration	Normal	0
	50 % increase	1		Moderate	1
	100 % increase	2		Severe	2
Maximum scores		10	Maximum scores		10

### Measurement of cytokines and MPO

The ileal tissue was grinded in liquid nitrogen, and homogenized in 1 ml phosphate buffered solution, pH 7.2 at 4 °C containing PMSF protein inhibitor. Homogenates were shaken at 60 Hz for 90 s, and the solution was centrifuged at 5000×*g* at 4 °C for 15 min. The supernatant was harvested. The protein concentrations were determined using a Nano Drop Spectrophotometer (Thermo Fisher Scientific, Inc., USA). Concentrations of TNF-α, IL-1β, IL-6 and MPO were measured using ELISA kits (Neo Bioscience Inc. Shenzhen, China for cytokines; eBioscience, Inc. USA for MPO) following the manufacturer’s instructions. Each sample was analyzed in triplicate and the values are presented as pg/mg.

### Statistical analysis

Experimental data are presented as the mean ± SD. Statistical analyses were performed using SPSS version 18.0 (SPSS Inc., Chicago, IL, USA). The statistical significant difference in macro- and micro-scopic scores between the groups was analyzed with the Mann–Whitney test. The statistical comparisons of other data were performed using independent *t* test. A difference was considered significant if P value was less than 0.05.

## Results

### TNBS dose determination for ileitis

Gross or microscopic lesions were not observed in the ileum of goats of the saline group. The TNBS-alone treated ileums showed minute neutrophil infiltration and increased wall thickness. The severity of ileal inflammation increased with the increase of TNBS dose (0–40 mg) in 40 % ethanol. The gross or microscopic scores in 30 mg TNBS in 40 % ethanol were 5.67 ± 0.57 and 6.33 ± 0.58, respectively and significantly higher (*P* < 0.05) than that in the 20 mg TNBS in 40 % ethanol group (3.33 ± 0.57 and 3.67 ± 0.58, respectively), but lower than that in 40 mg TNBS in 40 % ethanol (8.00 ± 1.00 and 8.67 ± 1.15, respectively). Therefore, 30 mg TNBS in 40 % ethanol induced a moderate ileitis and was chosen for further model experiments.

### Effect of TNBS treatment on body weight

**Fig. 2:**
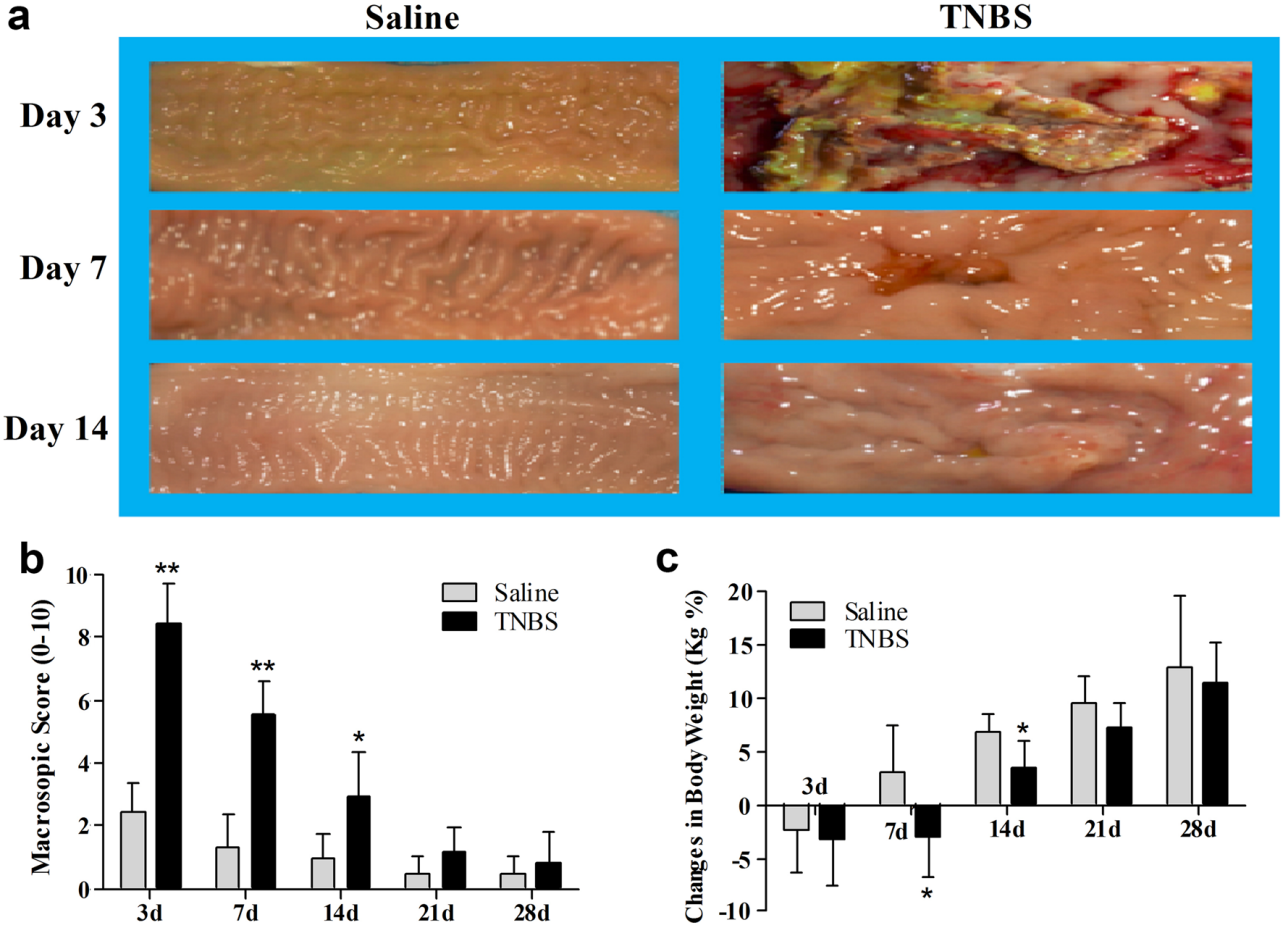
Morphologic changes (**a**), macroscopic scores (**b**) and body weight changes (**c**) of the TNBS-treated ileum in goats. TNBS group showed severe inflammation, ulceration and wall thickening at day 3, considerable damage in ileum at day 7, severity of these damages were decreased at day 14. The values are mean ± SD, n = 6. *P < 0.05 and **P < 0.01 show significant difference compared with the control

The saline-treated goats showed a decrease in body weight at day 3. The body weight of the TNBS-treated goats decreased at days 3 and 7. But both groups increased body weight at days 14 to 28. Compared with the saline group, body weight in the TNBS group was lower (*P* = 0.028 and 0.021) at days 7 and 14. There was no difference (*P* = 0.12 and 0.622) in body weight between two groups at days 21 and 28 (Fig. 2c).

### Macroscopic and microscopic changes of the TNBS-treated ileum

Gross lesions were not observed at days 3 to 21 after ileal saline injection. In the TNBS group, the ileal mucosa showed apparent congestion, hemorrhage, necrosis and wall thickness with widespread pseudo-membrane adhered to the mucosa at day 3, and light red color, sporadic adhered pseudo-membrane, localized necrosis and apparent wall thickening at day 7, and mild congestion and wall thickening at day 14 (Fig. 2a). No apparent lesions were observed in the adjacent visceral organs and tissues such as large intestines, jejunum and mesentery. Macroscopic lesion scores in the TNBS-treated ileum at days 3, 7 and 14 were 8.43 ± 1.29, 5.54 ± 1.11 and 2.90 ± 1.50, respectively and significantly more severe (*P* < 0.05) than those in the saline group (2.45 ± 0.99, 1.33 ± 0.98 and 0.95 ± 0.79, respectively). However, there was no difference (*P* > 0.05) in macroscopic lesion scores between the saline and TNBS treatments at days 21 and 28 (Fig. 2b).

**Fig. 3:**
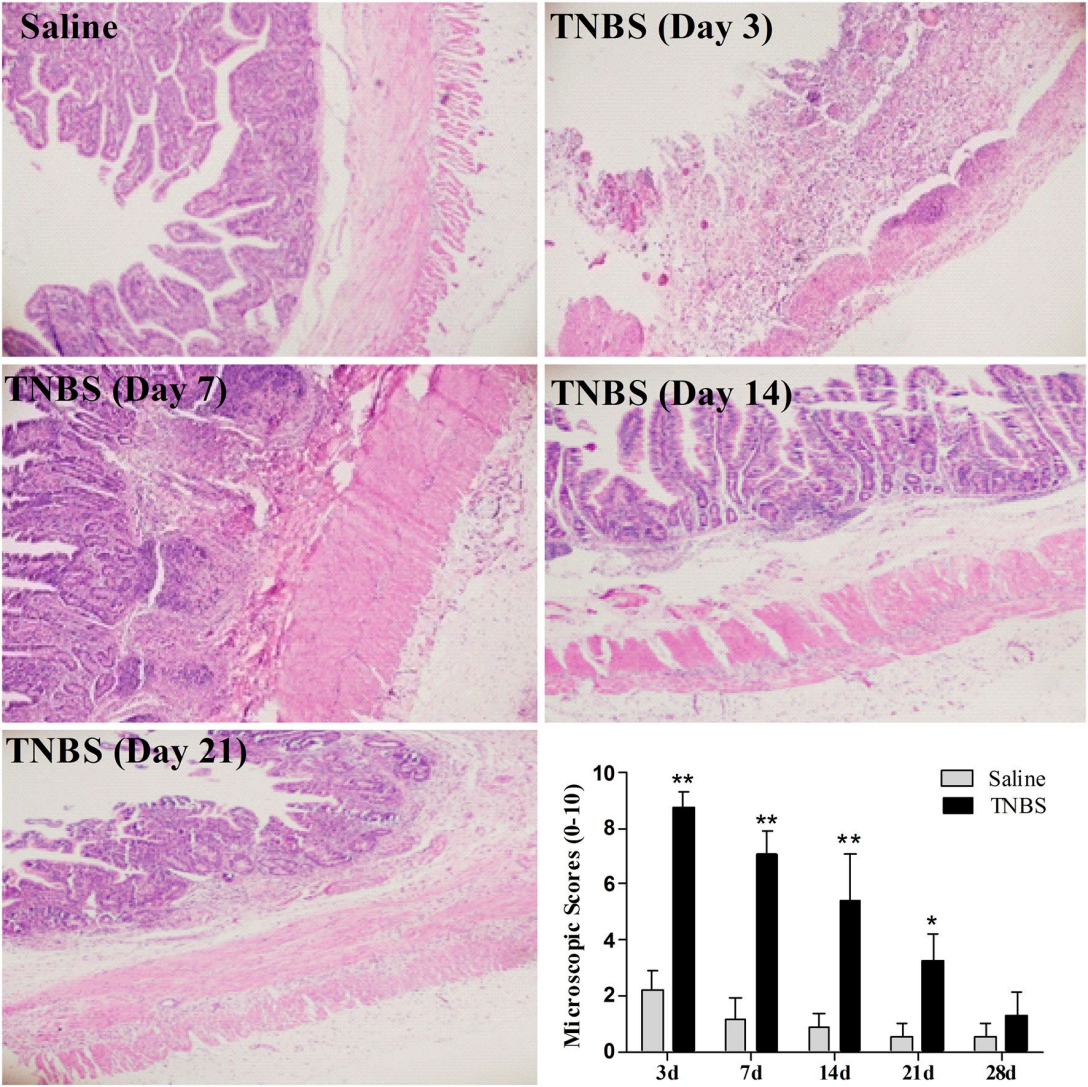
Microscopic lesions and microscopic lesion scores of the TNBS-treated ileum in goats. No significant inflammation in saline group, an extensive disruption of mucosal and submucosal layers, and inflammatory cell infiltration at day 3, ulceration of submucosal and muscular layers and inflammatory cell infiltration at day 7, Extensive inflammatory cells infiltration and granulomas at day 14, moderate infiltration of inflammatory cells and granulomas at 21 day after TNBS administration. The values are mean ± SD, n = 6. *P < 0.05 and **P < 0.01 show significant difference compared with the control

**Fig. 4:**
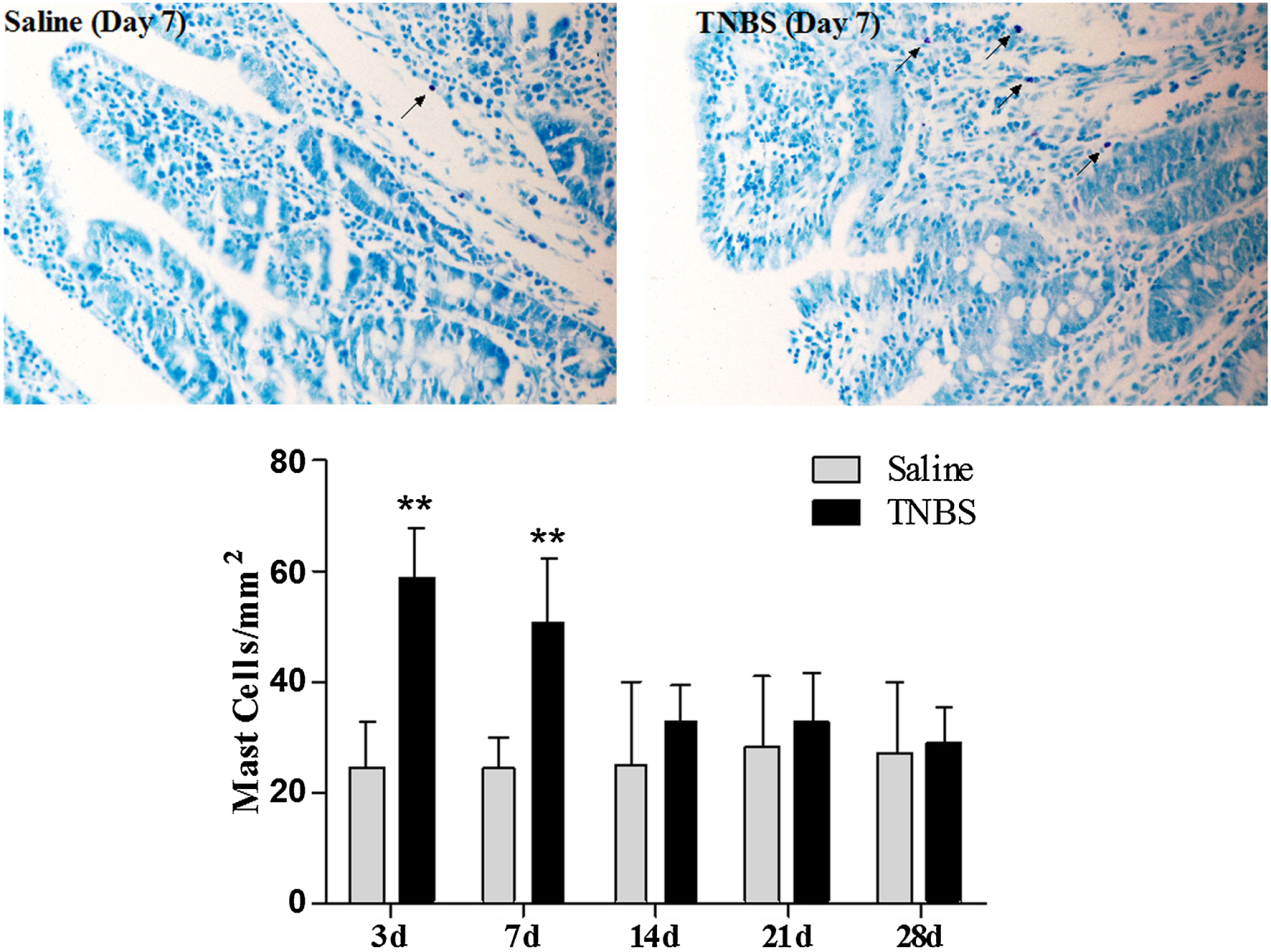
Mast cells in the ileal mucosa and mast cells count of the TNBS-treated ileum in goats. Mast cells were stained with 0.5 % toluidine blue (×200). Mast cells in saline group and in TNBS group at day 7 after treatments. The values are mean ± SD, n = 6 **P < 0.01 shows significant difference compared with the control

In the saline-treated goats, there were minute infiltrated inflammatory cells at day 3, but no inflammatory changes at days 7 to 28. In the TNBS group, the ileal wall showed apparent inflammatory cells infiltration and ulceration at day 3, extensive inflammatory cells infiltration, and apparent submucosal and muscular layer ulceration and blood vessel congestion at day 7, extensive inflammatory cells infiltration and granuloma at day 14, and moderate inflammatory cells infiltration and granuloma in the submucosa and muscular layer at day 21. Microscopic lesions were not observed in ileum at day 28 after TNBS treatment. Microscopic lesion scores in the TNBS group at days 3, 7, 14, and 21 were 8.78 ± 0.78, 7.11 ± 0.86, 5.41 ± 1.77 and 3.25 ± 0.52 and therefore significantly more severe (*P* < 0.05) than those of the saline group (2.17 ± 0.75, 1.16 ± 0.75, 0.88 ± 0.61 and 0.5 ± 0.54, respectively). However, there was no difference (*P* > 0.05) in microscopic lesion scores between the two groups at day 28 (Fig. 3). The number of mast cells in the ileum of the TNBS-group at days 3 and 7 were 58.63 ± 9.30 and 50.62 ± 11.48 per mm^2^, compared to 24.33 ± 8.27 and 24.51 ± 5.51 in the saline group (*P* < 0.01). However, there was no difference (*P* > 0.05) in mast cell counts between the saline and TNBS treatments at days 14 to 28 (Fig. 4).

### Concentrations of MPO and cytokines in the ileal tissue

**Fig. 5:**
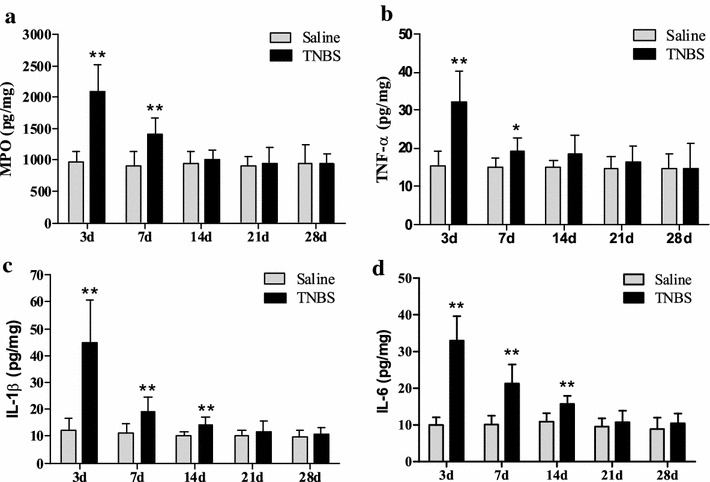
Measurement of MPO (**a**) and cytokines level like TNF-α (**b**), IL-1β (**c**) and IL-6 (**d**), and in ileum after TNBS administration (mean ± SD, n = 6). *P < 0.05 and **P < 0.01 show significant difference compared with the control

In the saline group, changes were not observed in levels of MPO, TNF-α, IL-1β or IL-6 throughout the experiment. The concentrations of MPO in the TNBS-treated ileum were 2089.03 ± 435.30 pg/mg at day 3 and 1406.27 ± 264.53 pg/mg at day 7, i.e. significantly higher than in the saline group (*P* < 0.01) (977.65 ± 168.47 and 913.47 ± 214.57 pg/mg, respectively). The MPO concentrations between two groups at days 14 to 28 did not differ (*P* > 0.05) (Fig. 5a). Compared with the saline group, concentrations of TNF-α, IL-1β and IL-6 in the TNBS group increased (*P* < 0.05) by 208.67, 364.72 and 330.15 % at day 3, and by 126.69, 168.71 and 209.60 % at day 7. The concentrations of IL-1β and IL-6 in the TNBS group increased (*P* < 0.05) by 140.22 and 143.93 % at day 14, respectively.

There were no differences (*P* > 0.05) in the concentrations of TNF-α at days 14 to 28 and IL-1β and IL-6 at days 21 to 28 between two groups (Fig. 5b–d).

### Visceromotor response

**Fig. 6:**
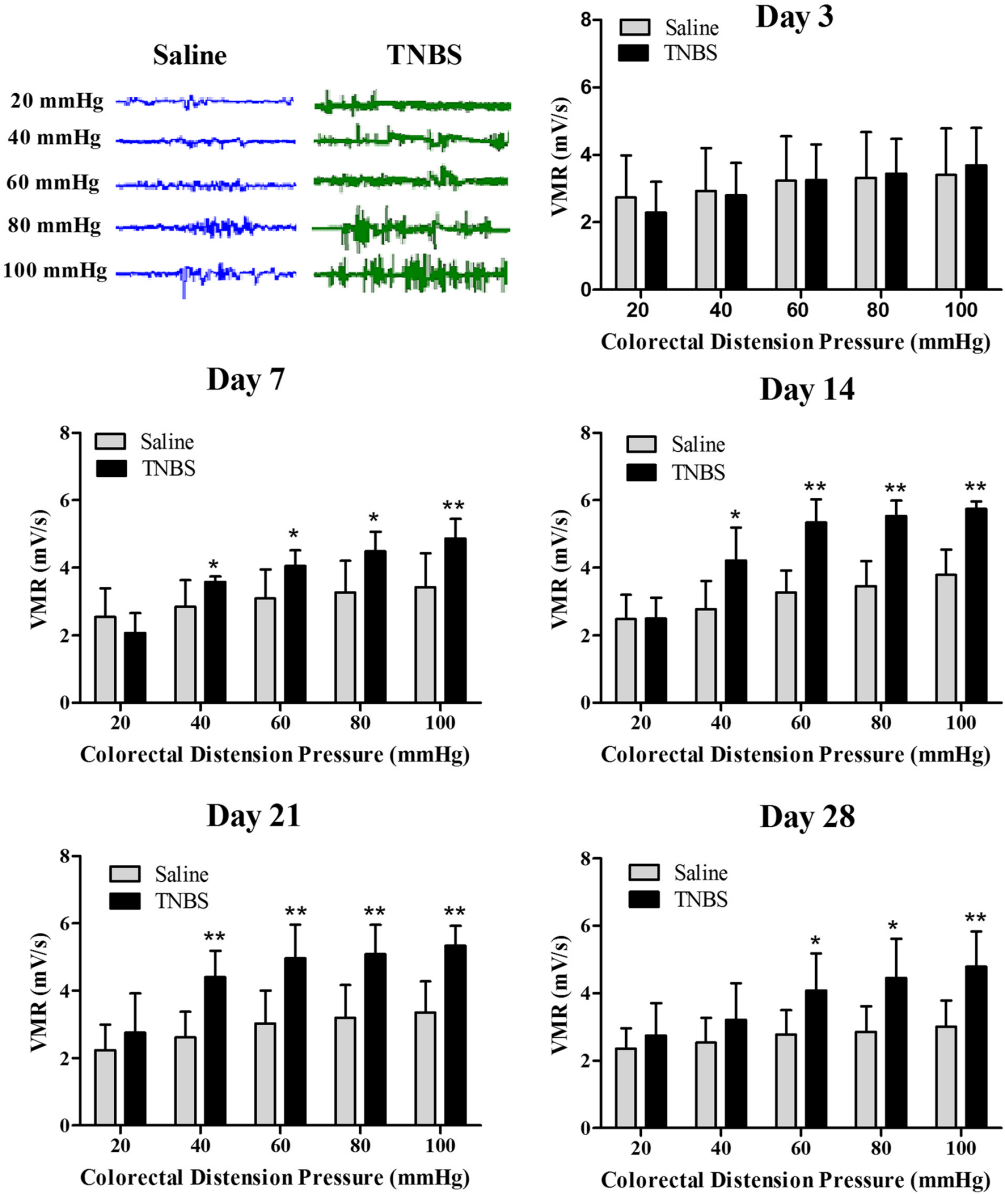
Representative electromyography (EMG) traces at day 14 and visceromotor responses (VMR) to colorectal distension (mean ± SD, n = 6) at days 3, 7, 14, 21 and 28 after treatments. *P < 0.05 and **P < 0.01 show significant difference compared with the control

Graded balloon distension (20, 40, 60, 80 and 100 mmHg) in colorectum resulted in VMR at days 7, 14, 21 and 28. The goats showed signs such as restlessness, rapid breathing, guarding, tail wagging, lips curling, head back to the abdomen and posture change when balloon distension was applied. There was no difference (*P* > 0.05) in VMR between the saline group and TNBS group at day 3. VMRs in the TNBS-treated goats were higher (*P* < 0.05) than those in the saline control in a pressure-dependent manner at days 7 to 28 except for that with 40 mmHg at day 28. Compared with the saline group, VMR in the TNBS group increased (*P* < 0.05) with 40, 60, 80 and 100 mmHg at day 7. VMR in the TNBS-treated goats increased (*P* < 0.05) with 40 mmHg, and reached greatest (*P* < 0.01) with 60, 80 and 100 mmHg at days 14 and 21. At day 28, VMR in the TNBS group increased (*P* < 0.05) with 60 and 80 mmHg, and further increased (*P* < 0.01) with 100 mmHg (Fig. 6).

## Discussion

Rectal instillation of TNBS-ethanol solution is an established model that is used to examine the pathogenesis of acute and chronic colitis, and to determine the mechanisms and efficacy of therapies. Some researchers have administered TNBS to induce ileitis. It seems that the inflammatory response of small intestine to TNBS is different from the well-characterized response of the colon. Most studies have shown that TNBS-induced ileitis lasts several days whereas the colitis persists for several weeks after TNBS administration. The administration and doses of TNBS differ between studies; the methodology is inconsistent, and no standardized protocol exists. Moreels et al. [22] injected 85 mg/kg body weight of TNBS in 40 % ethanol into rat’s ileum and found a marked inflammatory reaction occurred at 36 h and lasted up to 7 days according to MPO concentration and macroscopic and microscopic pathologic changes. O’Hara et al. [23] injected 60 mg/kg body weight of TNBS in 30 % ethanol into the ileum of guinea pigs and observed a severe inflammation at day 3. Nurgali et al. [24] translumenally administered 30 mg/kg body weight TNBS in 30 % ethanol in guinea pigs and observed the clinically as well as histologically inflammatory evidences up to 7 days. Merritt et al. [16] injected TNBS-ethanol solutions (0.5–1 g TNBS in 33–75 % ethanol) into the ileal lumen of pigs, and induced ileitis up to 7 days. These studies showed a transient ileitis induced by TNBS in these animals.

Most researchers have administered TNBS-ethanol solution (30 to 150 mg/kg body weight or 10 to 40 mg/rat of TNBS in 30 to 50 % ethanol) into the intestinal lumen to induce colitis or ileitis in rats. Studied showed that 50 % ethanol or 30 mg TNBS alone induced mild colitis for <7 days while 10 to 30 mg TNBS in combination with ethanol (30 or 50 %) caused mild to severe colitis for over 21 days in rats [25, 26]. We injected different TNBS-ethanol solutions (20, 30 and 40 mg TNBS in 40 % ethanol) into the ileal wall of goats, provoked mild to severe ileitis. It is believed that TNBS and ethanol act in concert on the intestinal tissue. TNBS as a hapten can bind intestinal tissue proteins, induce inflammatory and immune responses, and result in VH [17, 27]. Ethanol induces acute inflammation and may contribute to TNBS diffusion. The action of TNBS-ethanol solution in the lumen of the intestines was influenced by many factors. Intestine emptying, mucus secretion and peristalsis may reduce the contact time of TNBS with the ileal mucosa, influence severity and duration of inflammation [16]. To avoid these influences, Czaja et al. [28] injected 4 % paraformaldehyde into the multiple sites of the ileal wall of pigs and found that apparent transient ileitis occurred at day 3. So far ileitis induced by TNBS injection into the ileal wall has not been reported. Our study showed that administration of TNBS-ethanol solution (30 mg TNBS in 40 % ethanol) in the ileal wall caused moderate ileitis of each goat, characterized by weight loss, diarrhea, and histopathologically apparent changes including extensive congestion, hemorrhage, mucosal necrosis, granulomas, diffuse infiltrated neutrophils and lymphocytes in lamina propia, and thickened wall. These symptoms and pathological changes are similar to JD. According to macroscopic and microscopic changes, the TNBS-induced ileitis in our experiment occurred at day 3, persisted till day 21 and disappeared at day 28.

JD usually occurs in ruminants, and its transmural lesions include thickened wall and granulomas in the small intestine, which is similar to human CD [3]. According to the CD study, microbial invasion and activation of immune response may have an important role in the development of these lesions [29]. Epidemiology suggests some relationship between gut flora and inflammatory granulomas. Ruminants have more bacteria in the cecum than other omnivorous and carnivorous animals. The granuloma development in the present study showed that enteric immune system was activated by a number of microorganisms existing in the ileum and proliferating under the pathological condition or their retrograding from the cecum.

Abdominal pain and discomfort are amongst the most frustrating symptoms for patients suffering from IBD. They not only occur during acute flares of inflammation but also do during remission [30, 31]. Adam et al. [17] intracolonally administered TNBS in Lewis rats, and found VH occurred at day 3, disappeared at day 14, and reappeared at day 28 to 42 after TNBS administration. Zhou et al. [18] used 20 mg TNBS in 50 % ethanol in the colon of Sprague-Dawley rats, observed VH at days 2 to 28. Feng et al. [32] injected TNBS into the colon lumen of mice, and observed VH at days 7 to 14. Shah et al. [20] injected TNBS into the ileal lumen of Sprague-Dawley rats, observed ileitis at day 3, followed by VH at days 7 to 21. The discrepancies in onset time and duration of VH above may be due to different inflammation locations and species. We injected TNBS into the ileal wall of goats, observed a significant VH at days 7 to 28, which is similar to the results of Shah et al. [20]. Because our experiment stopped at day 28, how long the VH lasted needs to be further confirmed.

A single visceral afferent fiber can span over ten spinal cord segments, send terminal branches throughout the superficial and deep dorsal horn, as well as project to the contralateral dorsal horn [33, 34]. Studies showed that VH from the colorectum is mediated via both thoracolumbar and lumbosacral spinal afferent pathways whereas VH from terminal ileum is mainly mediated via thoracolumbar spinal afferent pathways [35–37]. Because there is a viscero-visceral convergence of nociceptive impulses from small and large intestines on the spinal neurons, CRD can be used as a nociceptive stimulator for ileum hypersensitivity. EMG is the most commonly used technique to quantify the abdominal muscle contractions in response to graded CRD.

Studies showed that in biopsies obtained from patients with IBDs, the number of mast cells was increased [38]. Shah et al. [20] found increased mast cells at day 3, followed by VH at day 7 after administration of TNBS into the ileal lumen of rats. Our study showed that administration of TNBS in the ileal wall induced increased mast cells at days 3 to 7, and VH at days 7 to 28 in goats, which is in consistency with the results of Shah et al. [20]. Ohashi et al. [39] injected TNBS into the proximal colon of wild rats and mast cell knockout rats, and found that TNBS induced VH in the wild rats, but not in the mast cell deficient rats. Studies have showed that TNBS-induced VH can be suppressed by an oral treatment with a mast cell stabilizer, doxantrazole, in a dose-dependent manner [40]. The activated mast cells are demonstrated to release inflammatory mediators (cytokines, protease etc.), thereby activate protease-activated receptor-2 (PAR-2). The activation of PAR-2 leads to sensitizing transient receptor potential vanilloid subtype-1 receptors and to trigger the release of substance-P (SP) and calcitonin gene-related peptide (CGRP), which ultimately elicits neurogenic inflammation and hypersensitivity [17, 41, 42]. These studies suggest an important role of mast cells in the initiation and development of VH.

Although the causes of VH is unknown, microbial activation of immune system and induction of pro-inflammatory cytokines such as IL-6, TNF-α and IL-1β seem to be critical in genetically predisposed individuals. Adam et al. [11] reported that VH occurred in Lewis rats with increased IL-6 level, but not in Fisher rats without increased IL-6 level. Deletion of the regulatory sequence consisting of adenosine-uracil multimers in the 3’untranslated region of cytokine-encoding transcripts in mice results in increased transcription and expression of TNF-α upon cell activation. These mice spontaneously develop a granulomatous CD-like disorder and an arthritis-like inflammation [43]. It is demonstrated that TNF-α triggers the subsequent release of IL-1β, which can produce hypersensitivity.

## Conclusions

Injection of 30 mg TNBS in 40 % ethanol into the terminal ileal wall of goats induced apparent inflammation and transmural lesions, which are morphologically very similar to JD and human CD. VMR results showed that VH occurred at day 7 after TNBS injection and lasted up to day 28. This experiment successfully constructed a more economic (compared with TNBS injection into the intestinal lumen) and reproducible ileitis and VH, which is useful for studying the pathogenesis of ruminant’s and human IBDs, and the mechanism underlying VH, and for evaluating the efficacy of new therapeutic regimens.

## Abbreviations

CD: Crohn’s disease
CRD: colorectal distension
ELISA: enzyme-linked immunosorbent assay
EMG: electromyography
IBDs: inflammatory bowel diseases
IL-1β: interleukin-1 beta
IL-6: interleukin-6
JD: Johne’s disease
MPO: myeloperoxidase
TNBS: 2,4,6-trinitrobenzenesulfonic acid
TNF-α: tumor necrosis factor-alpha
VH: visceral hypersensitivity
VMR: visceromotor response
